# Mechanism of the SNARC Effect in Numerical Magnitude, Time Sequence, and Spatial Sequence Tasks: Involvement of LTM and WM

**DOI:** 10.3389/fpsyg.2018.01558

**Published:** 2018-08-21

**Authors:** Qiangqiang Wang, Mowei Liu, Wendian Shi, Jingmei Kang

**Affiliations:** ^1^Department of Psychology, Shanghai Normal University, Shanghai, China; ^2^Department of Psychology, Trent University, Peterborough, ON, Canada; ^3^School of Psychology, Northeast Normal University, Changchun, China

**Keywords:** SNARC effect, numerical magnitude, time sequence, spatial sequence, mental number line, mental whiteboard hypothesis

## Abstract

The spatial-numerical association of response codes (SNARC) effect refers to the phenomenon that responses involving small numbers are faster with the left hand and responses involving large numbers are faster with the right hand. Previous studies have investigated the mechanism of the SNARC effect only when the time sequence is induced by centrally presented successive numbers. No study has investigated the mechanism of the SNARC effect when the spatial sequence is induced. Given that the induction of a spatial sequence together with a time sequence provides a new temporary reference for the serial order to be coded in working memory (WM), it would be interesting to examine the SNARC effect when both the time sequence and spatial sequence are induced. Therefore, a novel priming paradigm that simultaneously induced the time sequence and spatial sequence was employed in the present study to investigate the mechanism of the SNARC effect. Specifically, the time sequence and spatial sequence were induced by presenting a series of self-paced successive numbers, centrally or in a left-to-right or right-to-left direction, on the screen. Following the presentation of successive numbers, the probe number was centrally presented on the screen and university students were asked to distinguish to which time sequence or spatial sequence the probe number belonged by pressing a specified key of a qwerty keyboard. The results indicated that (1) the SNARC effect simultaneously appeared in the processing of the number magnitude and time sequence when only the time sequence was induced. (2) The SNARC effect disappeared in the processing of the time sequence; however, the SNARC effect coexisted in the processing of the numerical magnitude and spatial sequence when the spatial sequence was induced and participants performed a time sequence relevant task. (3) The SNARC effect coexisted in the processing of the numerical magnitude, time sequence, and spatial sequence when the spatial sequence was induced and participants performed a spatial sequence relevant task. Based on these results, we conclude that whether the SNARC effect coexists in the processing of the numerical magnitude, the time sequence and spatial sequence were influenced by the spatial sequence and relevant task. The results further support the mental whiteboard hypothesis and extended the WM account. Implications for theories on the SNARC effect were discussed.

## Introduction

### The SNARC Effect

When participants were instructed to perform the task of classifying random Arabic numbers presented on a screen by pressing the left or right key of a keyboard with their left or right hand, they responded faster to small numbers with the left hand and faster to large numbers with the right hand, regardless of the classification standard that was set (numerical magnitude or numerical parity). This phenomenon is referred to as the spatial numerical association of response codes (SNARC) effect, and it was first identified by Dehaene et al. (1990, 1993). Subsequent studies suggest that the SNARC effect can be observed in tasks that involve not only Arabic numbers (Dehaene et al., 1993; Nuerk et al., 2011; Tan and Dixon, 2011) but also non-numerical quantities (Ishihara et al., 2008; Holmes and Lourenco, 2011; Cho et al., 2012; Kirjakovski and Utsuki, 2012; Fischer et al., 2013; Fumarola et al., 2014). For example, Fumarola et al. (2014), directly and indirectly, examined the association between the side of response and luminance. A SNARC-like effect was identified in which faster responses were associated with the left hand for dark stimuli and the right hand for light stimuli (Fumarola et al., 2014). Moreover, the SNARC effect was identified in tasks that involved serial order (Gevers et al., 2003, 2004; Dodd et al., 2008; Previtali et al., 2010; Jonas et al., 2011). For example, when participants were instructed to respond to a serial order (e.g., letters of an alphabet, months of a year), they responded faster to an early sequential position with the left hand and faster to a later sequential position with the right hand (Gevers et al., 2003, 2004). In addition, Previtali et al. (2010) instructed participants to respond to a correct sequence with key pressing after learning and memorizing a series of successive pictures. Participants responded faster with the left hand to the pictures that were early in the original sequence and faster with the right hand to the pictures that were later in the original sequence (Previtali et al., 2010). To explain the SNARC effect, two cognitive mechanisms have frequently been discussed, namely, the mental number line (MNL) in long-term memory (LTM) and working memory (WM) account.

### LTM and the SNARC Effect

It was argued that the numbers were represented on the MNL from left to right according to the numerical magnitude (Restle, 1970; Dehaene et al., 1993). This MNL, which relates to LTM, is responsible for eliciting the SNARC effect. The MNL account was well supported by research on numerical and non-numerical quantities (Dehaene et al., 1993; Ishihara et al., 2008; Holmes and Lourenco, 2011; Nuerk et al., 2011; Tan and Dixon, 2011; Cho et al., 2012; Kirjakovski and Utsuki, 2012; Fischer et al., 2013; Fumarola et al., 2014). Furthermore, the left–right biases were neurologically supported and were observed in pre-literate children (Keus et al., 2005; Wood et al., 2008; Opfer et al., 2010; Opfer and Furlong, 2011; Zhao et al., 2012; Yang et al., 2014). It should be noted that the spatial orientation of the MNL is associated with the directionality of the writing system. For example, Arabic participants who used only a right-to-left writing system displayed a reverse SNARC effect, which suggests the MNL has a right-to-left directionality (Zebian, 2005; Fischer et al., 2009; Shaki et al., 2009; Shaki and Gevers, 2011).

Although the MNL account was largely supported, researchers reported that the SNARC effect could be reversed or disappeared (Bächtold et al., 1998; Ristic et al., 2006; Lindemann et al., 2008; Shaki et al., 2009; Abrahamse et al., 2016). For example, when participants were instructed to imagine numbers as time on a clock-face, small numbers induced faster responses for the right hand, and large numbers induced faster responses for the left hand (Bächtold et al., 1998). In addition, Prpic et al. (2016) recently examined whether visually presented note values, which are represented as a decreasing left-to-right progression, could produce a SNARC-like effect. They determined that when note values were task relevant, participants responded faster to large note values with the left key and vice versa. By contrast, when note values were task irrelevant, the direction of this association was reversed (Prpic et al., 2016). These findings suggest that the MNL account alone cannot sufficiently explain the SNARC effect. Therefore, scholars have attempted to explain the SNARC effect with a WM account.

### WM and the SNARC Effect

Although the LTM-dependent MNL plays an important role in the origination of the SNARC effect, recent studies have indicated that the serial order in WM could also activate the SNARC effect (Fischer et al., 2010; Treccani and Umiltà, 2011; Lonnemann et al., 2013; van Dijck et al., 2013; Ginsburg and Gevers, 2015; Huber et al., 2016; Ginsburg et al., 2017). For example, in one study, five self-paced successive numbers that ranged from 1 to 10 were centrally and randomly presented on the screen in the learning section. Participants were instructed to memorize these successive numbers in the order of presentation. In the test section, a go/no-go paradigm was used in which participants were presented all 10 numbers; however, they were instructed to only respond to the numbers presented in the learning section by judging the parity of them. Numbers that were not previously presented should be ignored. The results indicated that retrieving early items of the presented number series facilitated a left hand response, whereas later items facilitated a right hand response. Similar results were also obtained when researchers used fruit and vegetable names in the task (van Dijck and Fias, 2011). This study was the first investigation to indicate a close link between serial order in WM and spatial processing (Abrahamse et al., 2014). The WM account of the SNARC effect in serial order tasks has been supported by many recent studies (Ginsburg and Gevers, 2015; Huber et al., 2016; Ginsburg et al., 2017). However, it must be emphasized that whether the SNARC effect exists simultaneously in the processing of magnitude and serial order is mediated by the task used. Specifically, the SNARC effect only appeared in the processing of serial order and not in numerical magnitude when the serial order was induced and participants were instructed to respond only to the numbers that were used to induce serial order (go/no-go task) (Fischer et al., 2010; van Dijck and Fias, 2011). By contrast, the SNARC effect could occur simultaneously in the processing of both numerical magnitude and serial order when participants were instructed to response to all probe numbers after the serial order was induced (go task) (Ginsburg and Gevers, 2015; Huber et al., 2016). For example, Ginsburg and Gevers (2015) induced serial order by centrally presenting a series of successive numbers and instructed participants to memorize these successive numbers in the correct order before performing a magnitude comparison task. They determined that the SNARC effect occurred simultaneously in the processing of numerical magnitude and serial order in the go task, whereas only the ordinal position effect was identified in the go/no-go task (Ginsburg and Gevers, 2015).

How is serial order processed within WM? According to the mental whiteboard hypothesis, in the processing of serial order, each item in a sequence is bound to a specific position marker. The conjunctions will be recalled in a later retrieval task (Abrahamse et al., 2014). Abrahamse et al. (2016) further proposed an extended WM account to explain the mechanism of the SNARC effect in the processing of both numerical magnitude and serial order. According to this hypothesis, individuals build an experience-based mental “work space” when dealing with verbal content. When processing a verbal serial order, the brain will work to bind the items to specific internal spatial templates. The bindings are conducted based on previous experiences so that every item is connected to a very specific and the most relevant spatial coordinate (Abrahamse et al., 2016). The use of the internal space is very creative and flexible according to these hypotheses (Henson, 1998; Botvinick and Watanabe, 2007; Abrahamse et al., 2014, 2016; Kalm and Norris, 2014; Darling et al., 2017).

### The Present Research

Serial order consists of both a time sequence (on a time dimension) and a spatial sequence (on a space dimension). Previous studies have investigated the mechanism of the SNARC effect only when the time sequence was induced by centrally presented successive numbers (Fischer et al., 2010; van Dijck and Fias, 2011; Ginsburg and Gevers, 2015; Huber et al., 2016; Ginsburg et al., 2017). No study has investigated the mechanism of the SNARC effect when the spatial sequence was induced. The use of space in WM is very flexible (Abrahamse et al., 2014, 2016; Viarouge et al., 2014; Darling et al., 2017). Thus, the induction of the spatial sequence together with the time sequence may provide a new temporary reference for serial order to be coded in WM; therefore, the serial order will be coded differently in WM compared to when only the time sequence is induced. It would be interesting to examine the SNARC effect when both the time sequence and spatial sequence are induced. To further unravel the mechanism of the SNARC effect when the spatial sequence is induced, we employed a novel paradigm which simultaneously induced the time sequence and spatial sequence trial-by-trial to investigate whether the SNARC effect would coexist in the processing of the numerical magnitude, time sequence, and spatial sequence.

The current study consisted of three main experiments. The first experiment adopted a task-relevant time sequence, in which the participants were required to judge the time sequence (before or after the medial digit) of a probe digit when the time sequence was induced by a centrally presented series of self-paced successive numbers that consisted of five Arabic numbers on a computer screen. The purpose of Experiment 1 was to replicate the results of the previous research to examine the feasibility of our novel paradigm. Previous research has indicated that the SNARC effect coexists in the processing of both numerical magnitude and serial order in go tasks (Ginsburg and Gevers, 2015; Huber et al., 2016). Thus, we assume that the SNARC effect could be observed in the processing of both numerical magnitude and time sequence simultaneously in Experiment 1.

The second experiment was designed to investigate whether the SNARC effect could occur in the processing of the numerical magnitude, time sequence, and spatial sequence simultaneously when participants were instructed to judge which time sequence (before or after the medial digit) the probe numbers belong to following the presentation of successive numbers with a left-to-right or right-to-left presentation direction. It should be noted that the successive numbers were presented from left to right or right to left (half left-to-right, half right-to-left) on the computer screen to induce both the time sequence and spatial sequence. Moreover, it is possible that the time sequence and spatial sequence might correspond to and confound each other in a single presentation direction. For example, in the presentation of the successive numbers 1–2–5–8–9 from the left to right direction, 1, 2 belong to before 5 in the time sequence corresponding to belong to left in the spatial sequence if only from the left to right direction was used. Thus, the reverse direction was also employed to control the confounding effect of a single direction presentation. Given the involvement of the spatial sequence could provide a new temporary reference for serial order coding in WM, we expect the WM-related SNARC effect would be affected when both the time sequence and spatial sequence were induced.

The third experiment was designed to further determine whether the SNARC effect occurs in parallel in the processing of the numerical magnitude, time sequence, and spatial sequence when participants were instructed to determine which spatial sequence the probe number belonged to instead of the time sequence used in the second experiment.

## Experiment 1

### Materials and Methods

#### Participants

The participants included 26 university students (6 males, 20 females) from Northeast Normal University, China. The average age was 23.12 (*SD* = 3 years, with a range from 18 to 29 years). All participants were right-handed and had normal or corrected-to-normal vision. They all exclusively used a left-to-right reading/writing direction. Participation in the study was completely voluntary. Informed consent was obtained prior to starting the experiment. Students who participated received bonus research marks toward their final grade in a psychology course. None of the students had recently participated in similar experiments. The experimental protocol was approved by the ethics committee of the Shanghai psychological society.

#### Stimuli and Apparatus

Stimuli consisted of five black Arabic numbers (1, 2, 5, 8, and 9) printed in Times New Roman font (72 in size). The five digits were presented in various sequences at the rate of 1 digit per second. The stimuli were presented on a 19” computer screen running at 1024 × 768 resolution, with a refresh rate of 60 Hz.

#### Task and Procedure

**FIGURE 1:**
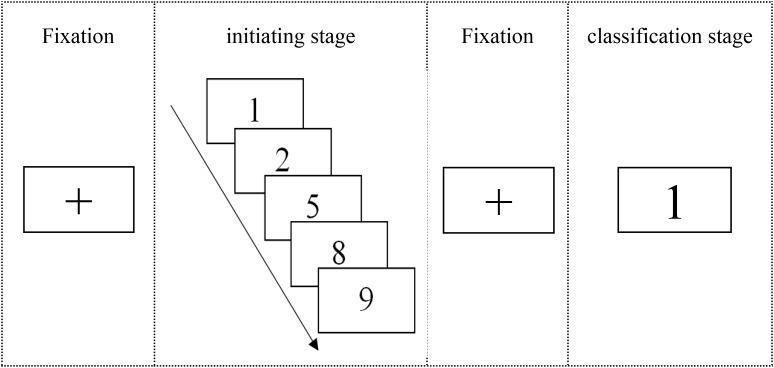
Example of a trial used in Experiment 1. Every trial comprised two stages: the initiating stage and the classification stage. There were four different sequences (1–2–5–8–9; 2–8–5–9–1; 8–9–5–1–2; and 9–1–5–2–8) presented in 192 trials, including 32 trials of practice.

The experiment was compiled with E-prime1.0 and was composed of two stages, including an initiating stage and a classification stage. In the initiating stage, a series of successive numbers that consisted of five Arabic numbers (e.g., 1–2–5–8–9) were continuously presented in the center of the computer screen at the frequency of one number per second in black type against a white background. Participants were instructed to memorize all digits in the correct order. A numerical sequence of five numbers was selected for the experiment because the adult memory capacity is 7 ± 2 blocks (Baddeley, 2012; Shipstead et al., 2015). In the classification stage, a random digit of the successive numbers learned in the initiating stage (with the exception of 5) was centrally presented on the screen, and the participants were instructed to distinguish in which time sequence the digit belonged to, before five or after five, by pressing a specified key of the qwerty keyboard as quickly and correctly as possible. Each stage of the test was initiated after a red “+” was centrally displayed on the computer screen for 500 ms, and the next trial was started approximately 1500 ms after the participants’ response (**Figure 1**). The respondents had 3 s to respond to each digit.

The successive numbers presented sequence was arranged as follows: 1–2–5–8–9; 2–8–5–9–1; 8–9–5–1–2; and 9–1–5–2–8. Among the five digits, five was always presented as the third number, whereas the remaining four numbers were presented in various sequences. Each of the remaining four numbers was presented in every sequential position with an equal frequency to eliminate interference caused by a difference in the numerical magnitude in each position of the sequential position.

The experiment was composed of two blocks. In the first block, the subjects were instructed to press the left key with the left index finger on the qwerty keyboard in response to digits before five in the memory sequence or the right key with their right index finger in response to digits after five in the memory sequence. The second block was the opposite (i.e., press the left key with their left index finger for digits after five in the memory sequence and the right key with their right index finger for digits before five in the memory sequence). The left and right keys corresponded to the “F” and “J” keys of the qwerty keyboard and were covered with stickers to prevent a potential confound caused by letters because letters of the alphabet are spatially coded similarly to numbers (Gevers et al., 2003). The two blocks were balanced between subjects. The entire experiment included 192 trials (160 trials of the formal experiment and 32 trials of practice) and lasted approximately 35 min. The subjects had a rest period after every 40 trials in the formal experiment.

### Results and Discussion

The overall error rate of the experiment was very low (*M* = 3.89%, range: 0.6–7.5%). Moreover, there was no speed–accuracy trade-off because the correlation between the reaction time and error was positive over all trials, *r*(26) = 0.48, *p* < 0.05. Therefore, no further error rate analyses were conducted. The mean response times (RTs) (excluding the RTs beyond three standard deviations of data from all trials, 3.94% of trials were excluded) were analyzed in an analysis of variance (ANOVA) with 2 [numerical magnitude: small (1, 2) vs. large (8, 9)] × 2 (time sequence: before 5 vs. after 5) × 2 (side of response: left key vs. right key) as within subject factors.

**FIGURE 2:**
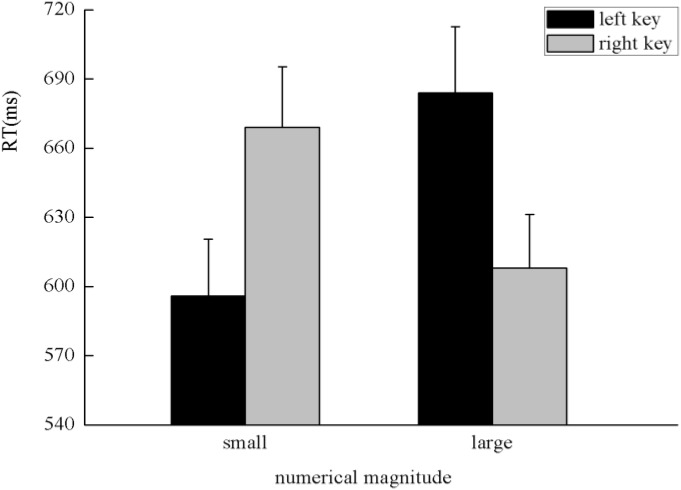
The SNARC effect was identified in the processing of numerical magnitude in Experiment 1, in which small numbers were responded to faster with the left key and large numbers were responded to faster with the right key.

**FIGURE 3:**
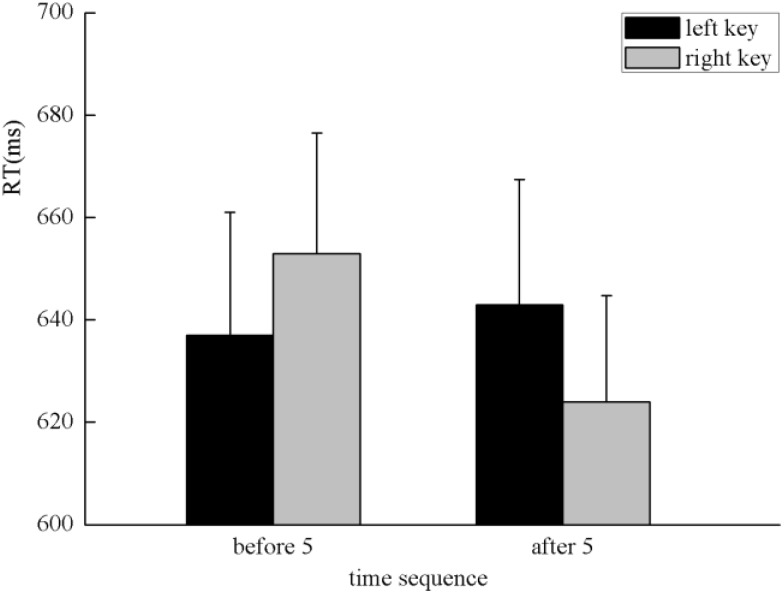
The SNARC effect was identified in the processing of time sequence in Experiment 1, in which digits before five were responded to faster with the left key and digits after five were responded to faster with the right key.

The results indicated that there was no significant main effect of numerical magnitude, time sequence, and side of response. A clear interaction between numerical magnitude and side of response was identified, *F*(1,25) = 5.80, *p* < 0.05, η^2^ = 0.188. A simple effect analysis showed that small numbers (596 ± 24.48 ms) were responded to faster than large numbers (684 ± 28.78 ms) with the left key, *F*(1,25) = 13.29, *p* < 0.01, η^2^ = 0.347. By contrast, large numbers (608 ± 23.20 ms) were responded to faster than small numbers (669 ± 26.67 ms) with the right key, *F*(1,25) = 5.84, *p* < 0.05, η^2^ = 0.189, which indicates the SNARC effect was identified in the processing of the numerical magnitude (**Figure 2**). Moreover, there was a significant interaction between the time sequence and side of the key, *F*(1,25) = 7.25, *p* < 0.05, η^2^ = 0.225. A simple effect analysis showed that digits before five (637 ± 24.02 ms) were responded to slightly faster than digits after five (643 ± 24.41 ms) with the left key, *F*(1,25) = 0.41, *p* > 0.05, η^2^ = 0.016. By contrast, digits after five (624 ± 20.78 ms) were responded to faster than digits before five (653 ± 23.52 ms) with the right key, *F*(1,25) = 7.30, *p* < 0.05, η^2^ = 0.226, which indicates the SNARC effect was identified in the processing of the time sequence (**Figure 3**). In addition, a significant interaction was identified between numerical magnitude and time sequence, *F*(1,25) = 12.17, *p* < 0.01, η^2^ = 0.327. A simple effect analysis showed a consistent effect that small numbers (631 ± 24.60 ms) were responded to slightly faster than large numbers (634 ± 24.46 ms) in the time sequence of before five, *F*(1,25) = 0.05, *p* > 0.05, η^2^ = 0.002, whereas large numbers (633 ± 22.23 ms) were responded to faster than small numbers (659 ± 23.34 ms) in the time sequence of after five, *F*(1,25) = 13.34, *p* < 0.01, η^2^ = 0.348. No significant interaction was identified among the numerical magnitude, time sequence, and side of response. In conclusion, the results of the first experiment indicated the SNARC effect appears simultaneously in the processing of the numerical magnitude and time sequence. This result replicated the results of previous studies (Ginsburg and Gevers, 2015; Huber et al., 2016). Thus, we deduce that our novel paradigm is feasible to induce serial order.

## Experiment 2

In Experiment 1, we proved our novel paradigm is feasible to induce serial order. Given that our novel paradigm can simultaneously induce time sequence and spatial sequence trial-by-trial, in Experiment 2, we simultaneously activated both the time sequence and spatial sequence by presenting successive numbers from left to right or right to left (the reverse direction was random and equal to prevent confounding with the involvement of a single presentation direction) and instructed subjects to judge whether the probe number centrally presented on the screen in the classification stage was presented before five or after five in the initiating stage to investigate whether the SNARC effect can coexist in the numerical magnitude, time sequence, and spatial sequence.

### Materials and Methods

#### Participants

The participants included 48 university students (17 males, 31 females) from Northeast Normal University, China. The average age was 21.79 (*SD* = 2.75 years, with a range from 18 to 28 years). All participants were right-handed and had normal or corrected-to-normal vision. They all exclusively used a left-to-right reading/writing direction. Participation in this study was completely voluntary. Informed consent was obtained prior to starting the experiment. Students who participated received bonus research marks toward their final grade in a psychology course. None of the participants had recently participated in a similar experiment. The experimental protocol was approved by the ethics committee of the Shanghai psychological society.

#### Stimuli and Apparatus

The stimuli and apparatus were the same as those used in Experiment 1.

#### Procedure and Task

The procedure used was similar to the procedure employed in Experiment 1 with the exception of the stimulus presentation pattern. In Experiment 2, the time sequence and spatial sequence were induced simultaneously by presenting a series of successive numbers in the vertical middle of a computer screen from left to right or right to left (the left-to-right or right-to-left direction was random and equal to control the confounding effect of a single direction presentation). The horizontal spatial position of each number was at 20, 35, 50, 65, and 80% of the screen width, respectively (the visual angle of the width compared to the screen edge was 4.53, 15.61, 21.75, 27.41, and 32.55° in approximately 47 cm of visual distance). The task of this experiment was to judge the time sequence; specifically, participants were instructed to judge whether the probe number centrally presented on the screen in the classification stage was presented before five or after five in the initiating stage.

### Results and Discussion

The overall error rate was very low (*M* = 3.11%, range: 0.39–10.94%). Moreover, there was no speed–accuracy trade-off because of an indistinctively positive correlation between the reaction time and error over all trials, *r*(48) = 0.27, *p* > 0.05. Thus, we did not further analyze the error rate in this experiment. The mean RTs (excluding beyond three standard deviations of data from all trials and incorrect trials, 5.47% of trials were excluded) were analyzed via an ANOVA with 2 [numerical magnitude: small (1, 2) vs. large (8, 9)] × 2 (time sequence: before 5 vs. after 5) × 2 (spatial sequence: left vs. right on the screen) × 2 (side of response: left key vs. right key) as within subject factors.

**FIGURE 4:**
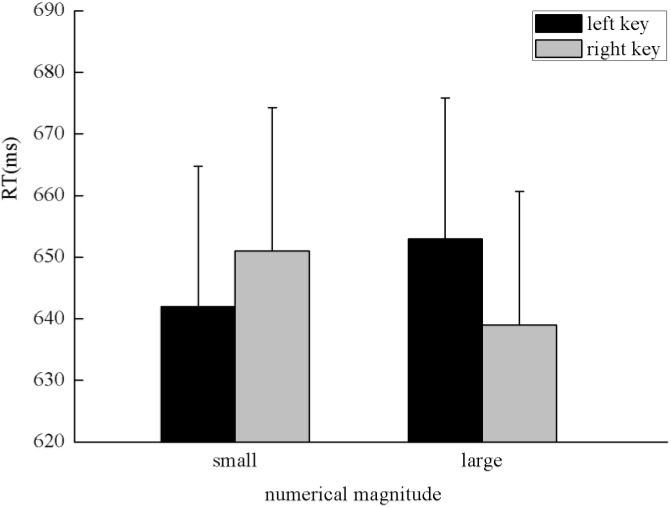
SNARC effect showing that small numbers were responded to faster with the left key and large numbers were responded to faster with the right key in the processing of numerical magnitude when participants were instructed to determine whether the specified stimuli were presented before five or after five in the initiating stage in Experiment 2.

**FIGURE 5:**
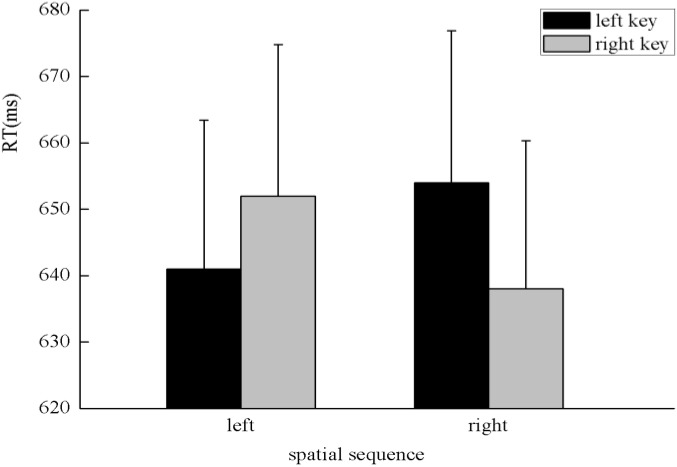
SNARC effect was observed in the processing of spatial sequence in this experiment. Specifically, the left key-pressing response was faster for digits on the left than digits on the right, and the right key-pressing response was faster for digits on the right than digits on the left.

There was a significant main effect in the time sequence, *F*(1,47) = 48.55, *p* < 0.001, η^2^ = 0.508, in which digits presented before 5 (617 ± 20.97 ms) were responded to faster than after 5 (675 ± 24.17 ms), which suggests a serial scanning strategy. There was a significant interaction between the numerical magnitude and side of response, *F*(1,47) = 4.64, *p* < 0.05, η^2^ = 0.09. A simple effect analysis showed a classical SNARC effect exhibited in the processing of the numerical magnitude, in which small numbers (642 ± 22.78 ms) were responded to slightly faster than large numbers (653 ± 22.85 ms) with the left key, *F*(1,47) = 2.04, *p* > 0.05, η^2^ = 0.042, and large numbers (639 ± 21.66 ms) were responded to faster than small numbers (651 ± 23.31 ms) with the right key, *F*(1,47) = 4.98, *p* < 0.05, η^2^ = 0.096 (**Figure 4**). There was a significant interaction between the spatial sequence and side of response, *F*(1,47) = 8.44, *p* < 0.01, η^2^ = 0.152. A simple effect analysis showed that digits presented on the left of the screen (641 ± 22.42 ms) were responded to faster than digits presented on the right of the screen (654 ± 22.89 ms) with the left key, *F*(1,47) = 5.47, *p* < 0.05, η^2^ = 0.104, and digits presented on the right of the screen (638 ± 22.34 ms) were responded to faster than digits presented on the left of the screen (652 ± 22.82 ms) with the right key, *F*(1,47) = 4.19, *p* < 0.05, η^2^ = 0.082; these findings indicate the SNARC effect was exhibited in the processing of the spatial position (**Figure 5**). A significant interaction was also identified between the numerical magnitude and time sequence, *F*(1,47) = 27.66, *p* < 0.001, η^2^ = 0.37. A simple effect analysis showed a consistent effect that numbers smaller than five (605 ± 20.52 ms) were responded to faster than numbers larger than five (688 ± 25.79 ms) in the time sequence of before five, *F*(1,47) = 61.26, *p* < 0.001, η^2^ = 0.566. By contrast, numbers larger than five (629 ± 21.70 ms) were responded to faster than numbers smaller than five (663 ± 22.97 ms) in the time sequence of after five, *F*(1,47) = 16.18, *p* < 0.001, η^2^ = 0.256. No other main effects or significant interaction effects were identified in this experiment.

The SNARC effect was not identified in the processing of the time sequence in Experiment 2. It is possible that the time sequence coded in WM is coded according to the spatial direction during encoding because the use of space in WM is very flexible (Abrahamse et al., 2014; Darling et al., 2017). As such, when analyzed together with the left–right condition, the effects may cancel each other out. Meanwhile, it is possible that the strong impact of numerical magnitude, and the interaction with serial order could obscure the chance of finding a time sequence SNARC effect depending on presentation direction, so that no time sequence could be observed. To test these possibilities, an ANOVA with 2 [numerical magnitude: small (1, 2) vs. large (8, 9)] × 2 (time sequence: before 5 vs. after 5) × 2 (presentation direction: left-to-right vs. right-to-left) × 2 (side of response: left key vs. right key) as within subject factors was conducted. There was a significant main effect in the time sequence, *F*(1,47) = 48.55, *p* < 0.001, η^2^ = 0.508, in which digits presented before five (617 ± 20.97 ms) were responded to faster than after five (675 ± 24.17 ms), which suggests a serial scanning strategy. There was a significant interaction between numerical magnitude and side of response, *F*(1,47) = 4.64, *p* < 0.05, η^2^ = 0.09. A simple effect analysis showed a classical SNARC effect exhibited in the processing of the numerical magnitude, in which small numbers (642 ± 22.78 ms) were responded to slightly faster than large numbers (653 ± 22.85 ms) with the left key, *F*(1,47) = 2.04, *p* > 0.05, η^2^ = 0.042, and large numbers (639 ± 21.66 ms) were responded to faster than small numbers (651 ± 23.31 ms) with the right key, *F*(1,47) = 4.98, *p* < 0.05, η^2^ = 0.096. There was a significant interaction between numerical magnitude and time sequence, *F*(1,47) = 27.66, *p* < 0.001, η^2^ = 0.37. A simple effect analysis showed a consistent effect that numbers smaller than five (605 ± 20.52 ms) were responded to faster than numbers larger than five (688 ± 25.79 ms) in the time sequence of before five, *F*(1,47) = 61.26, *p* < 0.001, η^2^ = 0.566. By contrast, numbers larger than five (629 ± 21.70 ms) were responded to faster than numbers smaller than five (663 ± 22.97 ms) in the time sequence of after five, *F*(1,47) = 16.18, *p* < 0.001, η^2^ = 0.256. Moreover, there was a significant interaction among time sequence, side of response, and presentation direction, *F*(1,47) = 8.44, *p* < 0.01, η^2^ = 0.152. A simple effect analysis indicated that there was no interaction between time sequence and side of response when successive numbers were presented from left to right, *F*(1,47) = 0.08, *p* = 0.779, η^2^ = 0.002, or when successive numbers were presented from right to left, *F*(1,47) = 1.66, *p* = 0.204, η^2^ = 0.034. However, the results indicated that there was a significant interaction between time sequence and presentation direction when responding to probe numbers with the left key, *F*(1,47) = 4.19, *p* < 0.05, η^2^ = 0.104, or when responding to probe numbers with the right key, *F*(1,47) = 5.47, *p* < 0.05, η^2^ = 0.082. A simple effect analysis showed that the digits presented before five (613 ± 20.94 ms) were responded to faster than its presented after five (675 ± 27.60 ms) with the left key in left–right condition, *F*(1,47) = 11.26, *p* < 0.01, η^2^ = 0.193, but the digits presented before five (632 ± 22.29 ms) were responded to slightly faster than its presented after five (669 ± 26.95 ms) with the left key in right–left condition, *F*(1,47) = 3.47, *p* = 0.069, η^2^ = 0.069. The digits presented before five (618 ± 22.23 ms) were responded to faster than its presented after five (672 ± 23.57 ms) with the right key in left–right condition, *F*(1,47) = 10.28, *p* < 0.01, η^2^ = 0.179, the digits presented before five (605 ± 24.60 ms) were responded to faster than its presented after five (686 ± 26.46 ms) with the right key in right–left condition, *F*(1,47) = 18.46, *p* < 0.001, η^2^ = 0.282. No other main effects or interaction effects were identified. In addition, to test whether the influence of numerical magnitude obscured the chance of finding a time sequence SNARC effect depending on presentation direction, for example, it is possible that the numerical SNARC would be present when presented successive numbers from left to right, overruling the time sequence SNARC, while in the right-to-left presentation direction, the numerical SNARC disappears, creating more chance of the time sequence SNARC to emerge. We further analyzed the SNARC effect of time sequence in each level of numerical magnitude and presentation direction. The results indicated that the SNARC effect of time sequence disappeared both in left–right condition and right–left condition, regardless of the numerical magnitude. Based on these results, we can eliminate the possibilities that the SNARC effect disappeared in the processing of the time sequence because analysis together with the left–right condition led to the effects that may cancel each other out and because of the influence of numerical magnitude.

Therefore, in Experiment 2, we conclude that the SNARC effect could not coexist in the numerical magnitude, time sequence, and spatial sequence; however, it coexisted in the numerical magnitude and spatial sequence when subjects were instructed to judge whether the digit centrally presented on the screen in the classification stage was presented before five or after five in the initiating stage.

## Experiment 3

In Experiment 2, we determined that the SNARC effect cannot coexist in numerical magnitude, time sequence, and spatial sequence when participants were instructed to judge whether the digit centrally presented on the screen in the classification stage was presented before five or after five in the initiating stage. The third experiment was designed to further investigate whether the SNARC effect coexists in the numerical magnitude, time sequence, and spatial sequence when participants are instructed to determine the spatial sequence instead of the time sequence used in Experiment 2.

### Materials and Methods

#### Participants

The participants included 46 university students (18 males, 28 females) from Northeast Normal University, China. The average age was 22.05 (*SD* = 3.14 years, with a range from 16 to 30 years). All participants were right-handed and had normal or corrected-to-normal vision. They all exclusively used to a left-to-right reading/writing direction. Participation in the study was completely voluntary. Informed consent was obtained prior to starting the experiment. Students who participated received bonus research marks toward their final grade in a psychology course. None of the students had recently participated in similar experiments. The experiment protocol was approved by the ethics committee of the Shanghai psychological society.

#### Stimuli and Apparatus

The stimuli and apparatus were the same as those used in Experiment 1.

#### Task and Procedure

The procedure used was similar to the procedure employed in Experiment 1 with the exception of the stimulus presentation pattern. In Experiment 3, the time sequence and spatial sequence were simultaneously induced by presenting a series of successive numbers in the vertical middle of a computer screen from left to right or right to left (the left-to-right or right-to-left direction was random and equal to control the confounding effect of a single direction presentation). The horizontal spatial position of each number was at 20, 35, 50, 65, and 80% of the screen width, respectively (the visual angle of the width compared to the screen edge was 4.53, 15.61, 21.75, 27.41, and 32.55° in approximately 47 cm of visual distance). Participants were instructed to judge whether the digit centrally presented on the screen in the classification stage was presented on the left or right space in the initiating stage in this experiment.

### Results and Discussion

The overall error rate was very low (*M* = 4.24%, range: 0.78–11.33%). Moreover, there was no speed–accuracy trade-off because of an indistinctively positive correlation between the reaction time and error over all trials, *r*(46) = 0.07, *p* > 0.05. Thus, we did not further analyze the error rate in this experiment. The mean RTs (beyond three standard deviations of data from all trials and all incorrect trials, 6.20% of trials were excluded) were analyzed via an ANOVA with 2 [numerical magnitude: small (1, 2) vs. large (8, 9)] × 2 (time sequence: before 5 vs. after 5) × 2 (spatial sequence: left vs. right on the screen) × 2 (side of response: left key vs. right key) as within subject factors.

**FIGURE 6:**
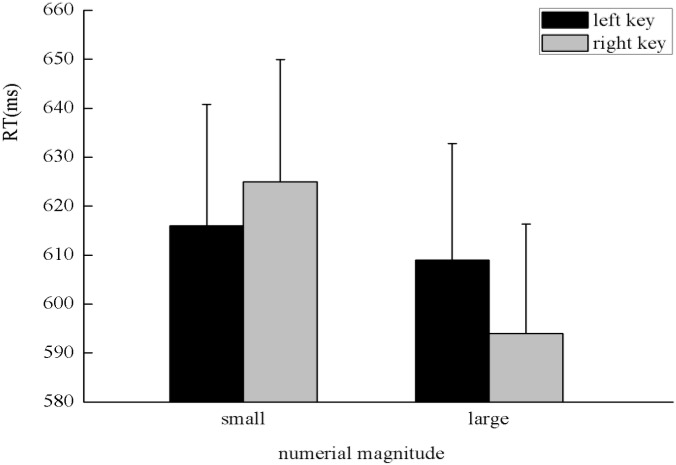
SNARC effect showing that small numbers were responded to faster with the left key and large numbers were responded to faster with the right key in the processing of numerical magnitude when participants were instructed to determine which portion of the screen the specified stimuli were presented in during the initiating stage in Experiment 3.

**FIGURE 7:**
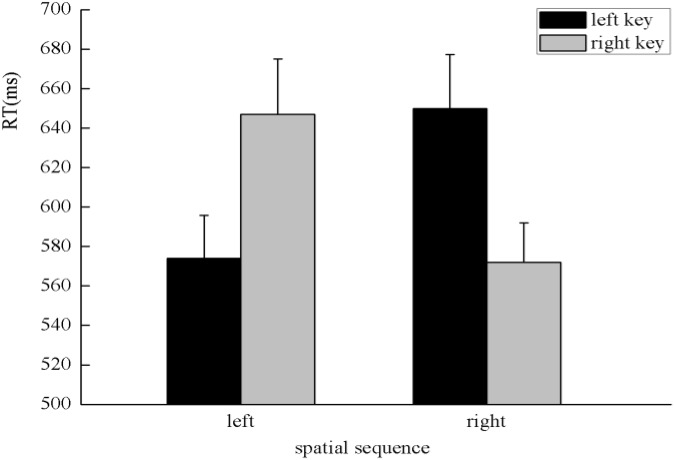
SNARC effect was exhibited in the processing of the spatial sequence, which demonstrated that participants responded faster with the left key to digits positioned on the left and responded faster with the right key to digits positioned on the right when subjects were instructed to determine which portion of the screen the specified stimuli were presented in the initiating stage in Experiment 3.

There was a significant main effect in the numerical magnitude, *F*(1,45) = 18.30, *p* < 0.001, η^2^ = 0.289, in which large numbers (601 ± 22.78 ms) were responded to faster than small numbers (621 ± 24.59 ms). There was a significant interaction between the numerical magnitude and side of response, *F*(1,45) = 5.88, *p* < 0.05, η^2^ = 0.116. A simple effect analysis showed a classical SNARC effect exhibited in the processing of the numerical magnitude, in which small numbers were responded to slightly faster with the left key (616 ± 24.77 ms) than the right key (625 ± 24.94 ms), *F*(1,45) = 1.78, *p* > 0.05, η^2^ = 0.038, and large numbers were responded to faster with the right key (594 ± 22.32 ms) than the left key (609 ± 23.76 ms), *F*(1,45) = 4.64, *p* < 0.05, η^2^ = 0.093 (**Figure 6**). There was a significant interaction between the spatial sequence and side of response, *F*(1,45) = 45.86, *p* < 0.001, η^2^ = 0.505. A simple effect analysis showed that digits presented on the left of the screen were responded to faster with the left key (574 ± 21.80 ms) than the right key (647 ± 28.02 ms), *F*(1,45) = 36.14, *p* < 0.001, η^2^ = 0.445, and digits presented on the right of the screen were responded to faster with the right key (572 ± 19.96 ms) than the left key (650 ± 27.40 ms), *F*(1,45) = 40.55, *p* < 0.001, η^2^ = 0.474, which indicates the SNARC effect was exhibited in the processing of spatial position (**Figure 7**). A significant interaction was identified between numerical magnitude and time sequence, *F*(1,45) = 9.68, *p* < 0.01, η^2^ = 0.177. A simple effect analysis showed a consistent effect that small numbers were responded to slightly faster in a time sequence of before five (615 ± 23.64 ms) than after five (626 ± 26.52 ms), *F*(1,45) = 1.08, *p* > 0.05, η^2^ = 0.024, whereas large numbers were responded to faster in a time sequence after five (591 ± 23.51 ms) than before five (611 ± 22.68 ms), *F*(1,45) = 7.20, *p* = 0.01, η^2^ = 0.138. No other main effects or significant interaction effects were identified in this experiment.

**FIGURE 8:**
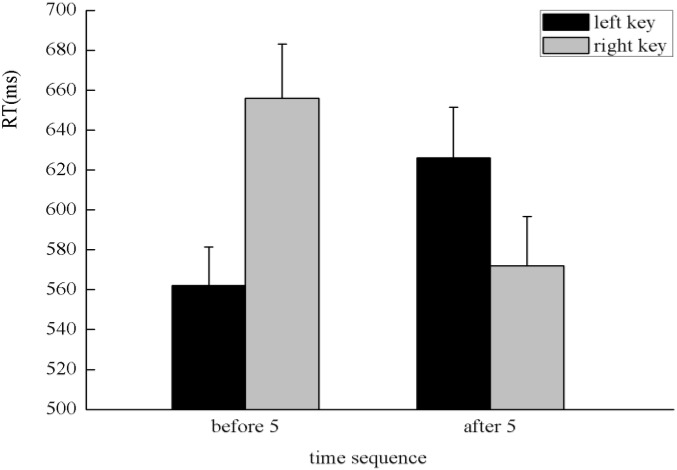
The classic SNARC effect was exhibited in the processing of the time sequence, which demonstrated that participants responded faster with the left key to digits before five and responded faster with the right key to digits after five when successive numbers were presented from left to right.

**FIGURE 9:**
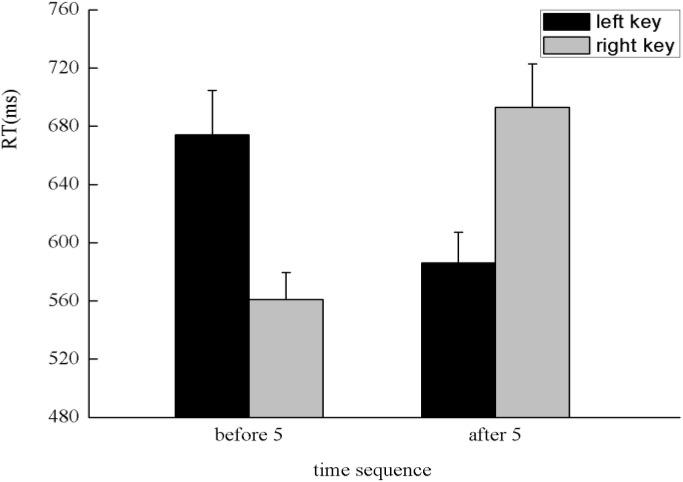
The reversed SNARC effect was identified in the processing of the time sequence, which demonstrated that participants responded faster with the left key to digits presented after five and responded faster with the right key to digits presented before five when successive numbers were presented from right to left.

Similar to Experiment 2, the SNARC effect was not exhibited in the processing of the time sequence in Experiment 3. It is possible that the SNARC effect disappeared in the processing of the time sequence because the effects may cancel each other out when analyzed together with the left–right condition or because of the strong impact of numerical magnitude and the interaction with time sequence. To test these possibilities, an ANOVA with 2 [numerical magnitude: small (1, 2) vs. large (8, 9)] × 2 (time sequence: before 5 vs. after 5) × 2 (presentation direction: left-to-right vs. right-to-left) × 2 (side of response: left key vs. right key) as within subject factors was conducted. There was a significant interaction among time sequence, side of response, and presentation direction, *F*(1,45) = 45.86, *p* < 0.001, η^2^ = 0.505. A simple effect analysis showed a significant interaction between the time sequence and side of response both in left–right condition, *F*(1,45) = 34.49, *p* < 0.001, η^2^ = 0.434, and right–left condition, *F*(1,45) = 41.14, *p* < 0.001, η^2^ = 0.478. A further simple effect analysis indicated that when successive numbers were presented from left to right, the digits presented before five were responded to faster with the left key (562 ± 19.43 ms) than the right key (656 ± 27.21 ms), *F*(1,45) = 42.06, *p* < 0.001, η^2^ = 0.483, and the digits presented after five were responded to faster with the right key (572 ± 24.76 ms) than the left key (626 ± 25.38 ms), *F*(1,45) = 15.71, *p* < 0.001, η^2^ = 0.259, which suggests a classic SNARC effect in the processing of the time sequence (**Figure 8**). When successive numbers were presented from right to left, the digits presented before five were responded to faster with the right key (561 ± 18.59 ms) than the left key (674 ± 30.58 ms), *F*(1,45) = 45.37, *p* < 0.001, η^2^ = 0.502, and the digits presented after five were responded to faster with the left key (586 ± 25.15 ms) than the right key (639 ± 29.82 ms), *F*(1,45) = 19.06, *p* < 0.001, η^2^ = 0.298, which suggests a reversed SNARC effect in the processing of the time sequence when successive numbers were presented from right to left (**Figure 9**). In addition, to test whether the numerical magnitude influenced the chance of finding a time sequence SNARC effect depending on presentation direction, we further analyzed the SNARC effect of time sequence in each level of numerical magnitude and presentation direction. The results indicated that the classic SNARC effect of time sequence appeared in the left–right condition, and the reverse SNARC effect appeared in the right–left condition, regardless of the numerical magnitude. Therefore, in Experiment 3, we concluded that the SNARC effect coexisted in the processing of the numerical magnitude, time sequence, and spatial sequence when participants were instructed to determine the spatial sequence instead of the time sequence.

## General Discussion

Previous studies have shown that whether the SNARC effect appears simultaneously in the processing of numerical magnitude and time sequence was mediated by the relevant task performed. Specifically, the SNARC effect could not coexist in the processing of serial order and magnitude in a go/no-go task (Fischer et al., 2010; van Dijck and Fias, 2011). For example, van Dijck and Fias (2011) reported that the SNARC effect disappeared in the processing of the numerical magnitude but not in the serial order when participants were instructed to judge the parity of probe numbers after memorizing a series of digits in the correct order. The SNARC effect of the numerical magnitude and time sequence could occur simultaneously in the go task (Ginsburg and Gevers, 2015; Huber et al., 2016). For example, Ginsburg and Gevers (2015) determined that the SNARC effect could result from the activation of both pre-existing positions of numbers at the MNL in LTM and the serial order in WM at the same time. Serial order consists of both the time sequence (on a time dimension) and spatial sequence (on a space dimension). Previous studies have investigated the mechanism of the SNARC effect only when the time sequence was induced by centrally presented successive numbers. No study has investigated the mechanism of the SNARC effect when the spatial sequence was induced. The induction of the spatial sequence together with the time sequence may provide a new temporary reference for serial order to be coded in WM; therefore, the serial order will be coded differently in WM compared to that coded when only the time sequence was induced. Thus, in the present study, we further investigated the mechanism of the SNARC effect when the spatial sequence was induced together with the time sequence. Specifically, we aimed to examine whether the numerical spatial representation on the MNL in LTM or the time sequence and spatial sequence in WM, independently or jointly, elicit the SNARC effect.

The numerical magnitude SNARC effect, which originated from the numerical spatial representation on the MNL in LTM, was identified throughout all three experiments in the present study through various tasks, including the task relevant time sequence and the spatial sequence. The results further support the viewpoint that the numerical magnitude SNARC effect could appear even when the numerical serial order was directly or indirectly activated (Dehaene et al., 1993; Ginsburg and Gevers, 2015; Huber et al., 2016). Our finding is inconsistent with several previous findings that suggest the numerical magnitude SNARC effect would disappear in a situation in which the serial order was activated (Fischer et al., 2010; van Dijck and Fias, 2011). The inconsistency may be a result of the design of the tasks. In the present study, similar to previous studies (e.g., Ginsburg and Gevers, 2015), a “respond all paradigm” was used in which participants were presented with a series of digits in the study stage. In the test stage, only the digits that were presented in the earlier study stage were used to test the potential SNARC effect (refer to Ginsburg and Gevers, 2015). By contrast, van Dijck and Fias (2011) explored the “go/no-go paradigm” in which in the test stage, participants were presented with digits that were more than what were learned in the study stage. Participants were instructed to only respond to the digits that were learned in the earlier study stage and ignore the digits that were not learned. In both designs, to complete the task in the test stage, participants should keep the memorized numerical sequence in mind while performing the required task. The mental representation of the learned tasks was more active and less interfered by the test in the “respond all paradigm” because what was tested was exactly what was learned. The direct relevance and lack of interference made retrieval substantially easier, which may, in turn, make the numerical magnitude SNARC effect observable.

In addition, not only was the numerical magnitude SNARC effect elicited in our three experiments but also the spatial sequence SNARC effect, which relates to WM. These findings suggest that the numerical spatial representation on the MNL in LTM and the spatial sequence in WM independently elicits the SNARC effect. The reason may be that subjects were provided with a numerical position marker, which may help solve the task (Abrahamse et al., 2014, 2016; Ginsburg et al., 2017). Thus, the numerical dimension was made more salient than the other dimensions, so it could be increased the chance to observe a numerical SNARC effect in our experiments. Unexpectedly, the SNARC effect was identified in the processing of the time sequence when a spatial sequence relevant task was performed, but not when a time sequence relevant task was performed, which suggests that whether the SNARC effect was observed in the processing of the time sequence was mediated by the relevant task performed. The reason why the SNARC effect disappeared in the processing of the time sequence when the time sequence relevant task was performed may be that the use of space in WM is very flexible (Abrahamse et al., 2014; Darling et al., 2017). When the time sequence relevant task was performed in Experiment 2, the time sequence relevant task involved horizontally arranged responses that favored a left to right encoding of the time sequence. At the same time, the left-to-right or right-to-left presentation direction provided a clear reference frame for the encoding of the time sequence. Therefore, when the time sequence relevant task was arranged, these two types of reference frames interacted with each other and lead the SNARC effect to disappear in the processing of the time sequence. However, when the spatial sequence relevant task was performed, it is possible that the time sequence coded in WM was coded according to the presentation direction during encoding. Thus, the classic SNARC effect was identified in the processing of the time sequence when successive numbers were presented from left to right, whereas the reversed SNARC effect was identified in the processing of the time sequence when successive numbers were presented from right to left in Experiment 3.

Recently, Abrahamse et al. (2016) proposed an extended WM account to explain the mechanism of the SNARC effect in the processing of numerical magnitude and serial order. They postulate that individuals build an experience-based mental “work space” when dealing with verbal content, including numerical magnitude and serial order (Abrahamse et al., 2016). According to the extended WM account, multiple item sets may be active in WM simultaneously and bias attention and/or response selection. In our experiments, when the spatial sequence relevant task was performed, the SNARC effect coexisted in the processing of both the time sequence and spatial sequence. This result further substantiated the extended WM account.

Furthermore, Abrahamse et al. (2014) proposed the mental whiteboard hypothesis on the basis of position marker models; they suggested that serial order coding in WM is very flexible. In our experiments, we determined that when the time sequence relevant task was performed, the SNARC effect disappeared in the processing of the time sequence. Moreover, when the spatial sequence relevant task was performed, the left-to-right presentation direction raised a classic SNARC effect, whereas the right-to-left presentation direction raised a reverse SNARC effect in the processing of the time sequence. Therefore, our findings also supported the mental whiteboard hypothesis. Furthermore, these findings illustrate that serial order coded in WM is coded according to the presentation direction during encoding. These findings simultaneously implied that the allocentric space is used in the spatial coding of serial order.

The SNARC effect widely exists in the processing of numerical magnitude and serial order; however, the mechanism may be different (Turconi et al., 2006; Zhao et al., 2012; Ginsburg and Gevers, 2015). We showed that regardless of the experimental task, the SNARC effect was identified in the processing of the numerical magnitude and spatial sequence in all situations. However, the SNARC effect could disappear in the processing of the time sequence when the spatial sequence was induced and the time sequence relevant task was performed, while a really strong SNARC effect of time sequence was observed when successive numbers were presented from left to right and time sequence was task irrelevant. Moreover, the SNARC effect could reverse in the processing of the time sequence when successive numbers were presented from right to left and the spatial sequence relevant task was performed. Based on these results, we conclude that the spatial encoding of the time sequence is more flexible than the numerical magnitude and spatial sequence. Thus, it is implied that the numerical magnitude, time sequence, and spatial sequence may have different processing mechanisms, and the spatial encoding of the time sequence was more flexible and easily affected by the experimental situation. Besides, in both Experiment 2 and in Experiment 3, there was no interaction between spatial sequence and numerical magnitude. There are a couple of studies showing that the physical location on the screen can also interact with numerical magnitude (Proctor et al., 2003; Notebaert et al., 2006). The results of this study were not consistent with previous studies, this can be an indication that this effect is related to response related processes. Meanwhile in all of three experiments, time sequence and numerical magnitude interact with each other. Furthermore, the numerical magnitude and time sequence interact more strongly with each other in the experiment where no time sequence SNARC was found. This suggests that some of the properties of both types of information are processed in a similar manner. On the other hand, the SNARC effect for numbers can be present in the absence of a SNARC effect for time sequence. Thus, where there are communalities in the processing, the spatial coding seems independent.

## Conclusion

In summary, we can draw the following conclusions from this study: (1) the SNARC effect simultaneously appeared in the processing of the number magnitude and time sequence when only the time sequence was induced. (2) The SNARC effect disappeared in the processing of the time sequence; however, the SNARC effect coexisted in the processing of the numerical magnitude and spatial sequence when the spatial sequence was induced and participants performed a time sequence relevant task. (3) The SNARC effect coexisted in the processing of the numerical magnitude, time sequence, and spatial sequence when the spatial sequence was induced and participants performed a spatial sequence relevant task. Based on these results, we conclude that whether the SNARC effect coexists in the processing of the numerical magnitude, the time sequence and spatial sequence were influenced by the spatial sequence and relevant task.

## Author Contributions

QW conducted the research under the guidance of WS and wrote the first draft of the manuscript. ML further modified and embellished the manuscript. JK gave strong support and help in the research.

## Conflict of Interest Statement

The authors declare that the research was conducted in the absence of any commercial or financial relationships that could be construed as a potential conflict of interest.
